# A Smartphone-Based M-Health Monitoring System for Arrhythmia Diagnosis

**DOI:** 10.3390/bios14040201

**Published:** 2024-04-18

**Authors:** Jun Luo, Mengru Zhang, Haohang Li, Dan Tao, Ruipeng Gao

**Affiliations:** School of Software Enginerring, Beijing Jiaotong University, Beijing 100044, China; 21301011@bjtu.edu.cn (J.L.); 21126367@bjtu.edu.cn (M.Z.); 20301132@bjtu.edu.cn (H.L.); dtao@bjtu.edu.cn (D.T.)

**Keywords:** arrhythmia diagnosis, ECG signal denoising, m-health service, deep learning

## Abstract

Deep learning technology has been widely adopted in the research of automatic arrhythmia detection. However, there are several limitations in existing diagnostic models, e.g., difficulties in extracting temporal information from long-term ECG signals, a plethora of parameters, and sluggish operation speed. Additionally, the diagnosis performance of arrhythmia is prone to mistakes from signal noise. This paper proposes a smartphone-based m-health system for arrhythmia diagnosis. First, we design a cycle-GAN-based ECG denoising model which takes real-world noise signals as input and aims to produce clean ECG signals. In order to train its two generators and two discriminators simultaneously, we explore an unsupervised pre-training strategy to initialize the generator and accelerate the convergence speed during training. Second, we propose an arrhythmia diagnosis model based on the time convolution network (TCN). This model can identify 34 common arrhythmia events using eight-lead ECG signals, and we deploy such a model on the Android platform to develop an at-home ECG monitoring system. Experimental results have demonstrated that our approach outperforms the existing noise reduction methods and arrhythmia diagnosis models in terms of denoising effect, recognition accuracy, model size, and operation speed, making it more suitable for deployment on mobile devices for m-health monitoring services.

## 1. Introduction

Cardiovascular disease has become a major global health threat, with sudden cardiac death being a significant cause of mortality. According to the World Heart Federation (WHF) World Heart Report 2023 [1], the number of deaths from cardiovascular disease increased from 12.1 million in 1990 to 18.6 million in 2019. In 2021, approximately 20.5 million people died from cardiovascular disease, accounting for about one-third of total global deaths. Arrhythmia, a common cardiovascular disease caused by abnormal cardiac electrical conduction, poses significant risks to human health. Even benign arrhythmias also indicate that the body’s heartbeat is irregular and may be potentially risky. Therefore, heart beat monitoring serves as a significant reminder for patients.

Given the increasing significance of cardiovascular health, real-time and continuous heart monitoring is imperative to avert potential accidents when individuals encounter cardiovascular diseases or related symptoms. Electrocardiogram (ECG) emerges as a safe, reliable, and noninvasive diagnostic method extensively employed in the clinical diagnosis and treatment of arrhythmia [2,3,4]. Nevertheless, the specialized medical knowledge required for arrhythmia diagnosis heavily relies on manual assessments, which are time-consuming and labor-intensive.

Currently, deep learning methods, such as convolutional neural networks (CNNs) [5] and recurrent neural networks (RNNs) [6], are widely employed for arrhythmia detection [7,8,9,10]. However, existing arrhythmia diagnosis models have certain limitations. CNN-based models always struggle to extract the time series features in ECG signals, which reflect the rhythm and regularity of cardiac activity which are crucial for arrhythmia diagnosis. While RNN-based models partially address this issue, their large number of parameters and inability to perform parallel operations make them unsuitable for mobile deployment. Therefore, developing an arrhythmia diagnosis model with high recognition accuracy, small model size, fast operation speed, and suitability for mobile terminal deployment is of great significance for the development of ECG monitoring at home.

Furthermore, unlike professional medical equipment, wearable m-health sensors are susceptible to various factors during data collection and transmission, resulting in noisy ECG signals. Such noise directly affects the accuracy of arrhythmia diagnosis. Existing ECG signal denoising methods are designed for specific types of analog noise, but real-world environments produce much complex noise. Consequently, effectively separating clean ECG signals from noisy sensory readings poses another challenge in this study.

This paper concentrates on designing and implementing an m-health system for ECG monitoring. Our contributions include:

- We propose an ECG denoising model based on cycle-GAN [11] to mitigate the impact of noise on arrhythmia diagnosis. The model employs a denoising autoencoder (DAE) structure as the generator, which enhances the noise reduction performance by adding analog noise to input signals. Experimental results have demonstrated that our approach outperforms the existing noise reduction methods.
- We devise an arrhythmia diagnosis model based on a time convolution network (TCN) to identify 34 common arrhythmia events [12] using eight-lead ECG signals. The model extracts effective healthcare features through two-dimensional convolution layers and parallel TCN modules and captures temporal information during long-term sequences. Experimental results have indicated that our approach surpasses existing arrhythmia diagnosis models in terms of recognition accuracy, model size, and operation speed.

This paper is organized as follows: Section 2 describes related work, Section 3 introduces our ECG noise reduction algorithm, Section 4 presents our arrhythmia diagnosis algorithm, Section 5 provides evaluation results, and Section 6 concludes the research.

## 2. Related Work

Traditional ECG signal denoising methods include adaptive filtering [13], empirical mode decomposition (EMD) [14,15], wavelet transform [16,17], and FIR filtering [18]. Among them, Rahman et al. [13] proposed a method using an adaptive filter based on error normalization to reduce ECG signal noise. This filter does not require a multiplier in the weight update process and exhibits good computational performance. Kabir et al. [14] proposed a windowing method integrated with EMD to remove noise from the initial intrinsic mode function (IMF). By performing windowing in the EMD domain, this method effectively reduces IMF noise while preserving the QRS complex, resulting in a cleaner ECG signal.

With the continuous development and maturation of deep learning technology, ECG signal denoising methods based on deep learning have also emerged [19,20,21,22,23,24]. These methods eliminate the need to distinguish noise types and can effectively remove noisy data. Among them, Qian Wei et al. [19] proposed a multi-layer noise reduction self-encoder method for ECG signal denoising. Peng et al. [20] introduced the use of stacked compression noise reduction self-encoders (CDAE) and an improved version of noise reduction self-encoder DAE to remove noise data from ECG signals.

Common traditional arrhythmia diagnosis methods include support vector machines (SVMs) [25], the k-nearest neighbor (KNN) algorithm [26], principal component analysis (PCA) [27], etc. Although these methods enable the automatic diagnosis of arrhythmia, they require manual feature extraction from ECG signals. Due to the temporal nature of ECG signals, many researchers have focused on using recurrent neural networks (RNNs) to extract ECG characteristics. The most commonly used RNNs are long short-term memory (LSTM) networks and gated recurrent units (GRUs). For instance, Georgios et al. [28] proposed a hybrid network composed of a CNN and LSTM for detecting atrial fibrillation. T. M. Ingolfsson et al. [29] presented an arrhythmia diagnosis algorithm based on the time convolution network (TCN) [30]. Shorda [31] utilized a CNN and bidirectional LSTM based on a residual network for arrhythmia classification. Cui Kaixing [32] and Hu Lin [33] constructed arrhythmia diagnosis models based on a CNN and LSTM.

Existing ECG monitoring systems have certain limitations. Firstly, most systems only collect single-channel or dual-channel ECG signals, neglecting the use of full-lead ECG signals, which limits the captured ECG information. Refs. [34,35] show that the 12-lead electrocardiogram (ECG) is a common method of recording the electrical activity of the heart, which uses multiple electrodes in different locations to record the heart’s electrical signals. In contrast, the single-lead ECG uses only one electrode to record the heart’s electrical activity. Obviously, doctors are able to observe the heart’s electrical activity in different directions at the same time with 12-lead ECGs, thus obtaining more comprehensive information, which helps them make more accurate diagnoses and treatments for heart lesions. Since the 12-lead ECG signals in III-lead, AVR-lead, AVL-lead, and AVF-lead can be derived from the other leads with linear operations, we select the 8-lead ECG signals to use in this paper. Secondly, some systems fail to consider the impact of ECG signal quality on diagnosis results. Household ECG sensors are susceptible to environmental interference and may collect noisy signals. Noisy ECG signals can adversely affect the diagnosis results of algorithms. Thus, a noise reduction strategy is necessary to improve signal quality and ensure reliable diagnosis results.

## 3. ECG Denoising Algorithm

This section introduces our ECG denoising algorithm. ECG signals reflect a person’s heart activity in a specific state, making it challenging to collect corresponding clean and noisy ECG data simultaneously. Current solutions involve manually adding simulated noise, but the limited types of simulated noise significantly differ from real-world noise.

**Figure 1:**
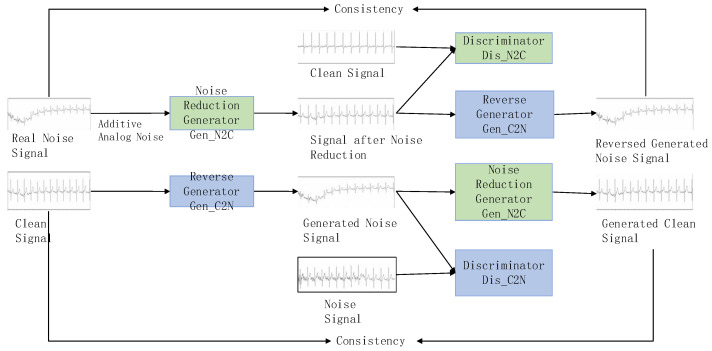
ECG noise reduction architecture.

To address this, we propose a cycle-GAN-based ECG signal denoising model, using noise from a real-world environment as input and aiming to produce clean ECG signals (shown in Figure 1). Two generators facilitate the conversion between noise and clean signals without requiring matching relationships in training data. Additionally, the generator is based on a denoising autoencoder (DAE) [36] to enhance noise reduction performance and model robustness by incorporating simulated noise into the real noise signal. Next, we delineate the comprehensive architecture of the denoising model, encompassing the structures of both the generator and discriminator.

The ECG signal denoising model specially trains two generators and two discriminators simultaneously to perform the conversion between noise signals and clean signals. The denoising generator (Gen_N2C) converts noise signals into clean signals, while the reverse generator (Gen_C2N) converts clean signals back into noise signals. The discriminator (Dis_N2C) and the discriminator (Dis_C2N) are used to judge whether the data generated by the two generators are close to the real data distribution. Through this cycle, the capabilities of the generators and discriminators are significantly improved, and finally it enhances the effectiveness of denoising.

The entire conversion process mainly includes four stages: adversarial training, reverse generation, cycle consistency training, and identity constraint training. In particular, adversarial training refers to the adversarial process between the generator and discriminator; reverse generation refers to the fact that the inputs and target outputs of the denoising generator (Gen_N2C) and the reverse generator (Gen_C2N) are opposite; cycle consistency training refers to the fact that the main content of the noise data remains unchanged after one cycle of conversion; and identity constraint training refers to the fact that the effective data is not affected after the noise-carrying signal is denoised.

**Figure 2:**
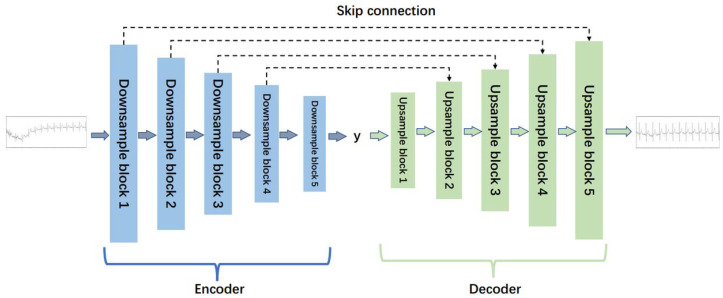
The structure of the generator for ECG denoising.

In the ECG signal denoising model, the denoising generator (Gen_N2C) and the reverse generator (Gen_C2N) have the same structure, both based on a denoising auto-encoder. Each generator includes an encoder and a decoder. The encoder encodes the input signal, extracts high-dimensional features, and the decoder reconstructs the data, mapping the feature values to the same dimension as the input signal. The specific structure of the generator is shown in Figure 2.

**Figure 3:**
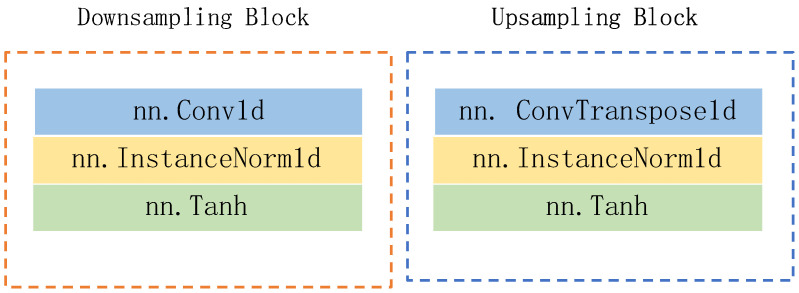
The structures of downsampling block and upsampling block.

As depicted in Figure 2, the generator adopts a five-layer noise reduction self-encoder structure. The encoder component comprises five lower sampling blocks, while the decoder component consists of five upper sampling blocks. Each subsampling block is constructed with a one-dimensional convolution layer (‘conv1d’), a regularization layer (‘instancenorm1d’), and an activation function layer (‘tanh’). Similarly, each upsampling block is comprised of a one-dimensional deconvolution layer (‘convtranspose1d’), a regularization layer (‘instancenorm1d’), and an activation function layer (‘tanh’). The detailed structures of the lower sampling block and the upper sampling block are illustrated in Figure 3.

**Figure 4:**
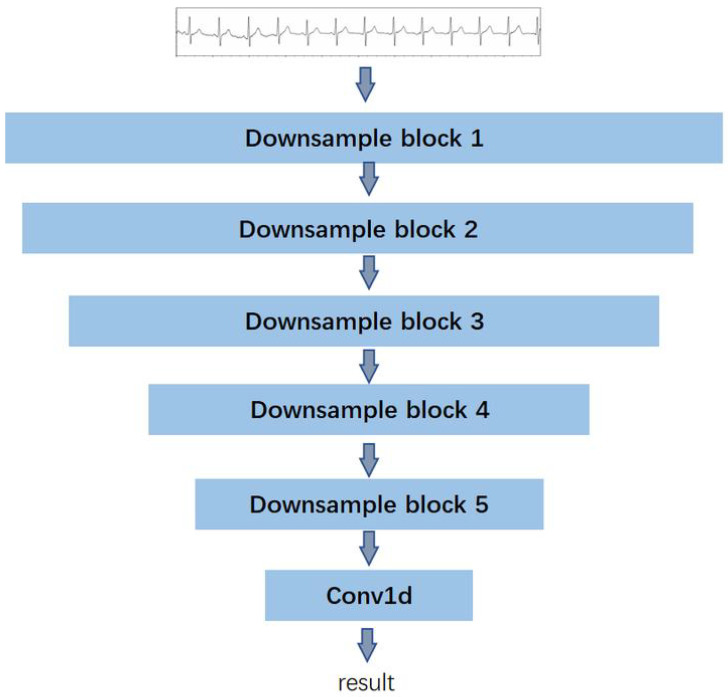
The structure of the discriminator for ECG denoising.

Corresponding to the generator, the structure of the two discriminators is identical. Each discriminator is constructed with five subsampling blocks and one one-dimensional convolution layer. The detailed structure is illustrated in Figure 4.

The total loss value comprises three components: the adversarial loss, the cycle consistency loss, and the identity loss. We present the calculation of loss function in Equation (1), i.e.,

(1) $$Loss=Loss_{GAN}+\alpha Loss_{cycle}+\beta Loss_{identity}$$

In particular, $Loss_{GAN}$ represents the adversarial loss and serves to assess the generation and discrimination capabilities of the generators and discriminators during the adversarial evolution. The objective of employing adversarial loss is to minimize the disparity between the distribution of real samples and the distribution of generated samples. This facilitates the generator in producing more authentic and clean ECG signals, while enabling the discriminator to more effectively distinguish real samples from the generated ones. Equation (2) presents its definition, i.e.,

(2) $$\begin{array}{cc} Loss_{GAN} & =Loss_{GAN}\left(G_{N2C},D_{N2C},N,C\right)+Loss_{GAN}\left(G_{C2N},D_{C2N},C,N\right)\\ \; & =E_{n∼Pdata(n)}\left[\log D_{N2C}\left(n\right)\right]+E_{C∼Pdata(c)}\left[\log\left(1-D_{N2C}\left(G_{N2C}\left(c\right)\right)\right)\right]\\ \; & +E_{c∼Pdata(c)}\left[\log D_{C2N}\left(c\right)\right]+E_{n∼Pdata(n)}\left[\log\left(1-D_{C2N}\left(G_{C2N}\left(n\right)\right)\right)\right] \end{array}$$

$Loss_{cycle}$ refers to the loss of cycle consistency, which is used to measure the difference between the data after two conversions and the original data. Its function is to maintain the consistency of the data during the cycle, that is, to ensure that the semantics and main contents of the original data remain unchanged after the ECG signal is denoised and re-converted, as Equation (3) shows:

(3) $$\begin{array}{c} Loss_{cycle}=E_{n∼Pdata(n)}\|G_{C2N}\left(G_{N2C}\left(n\right)\right){-n\|}_{1}+E_{C∼Pdata(c)}{\left\|G_{N2C}\left(G_{C2N}\left(c\right)\right)-c\right\|}_{1} \end{array}$$

Finally, $Loss_{identity}$ represents the identity loss. Its primary function is to ensure that the generator does not alter the main characteristics of the input image. In other words, it aims to minimize the difference between the input and output of the generator, ensuring that the generated signal retains authenticity and preserves essential information. Equation (4) presents its definition:

(4) $$\begin{array}{c} Loss_{identity}=E_{n∼Pdata(n)}\|G_{C2N}{\left(n\right)-n\|}_{1}+E_{C∼Pdata(c)}{\left\|G_{C2N}\left(c\right)-c\right\|}_{1} \end{array}$$

In Equations (2)–(4), $G_{N2C}$ and $G_{C2N}$ represent the noise reduction generator and reverse generator, respectively, $D_{N2C}$ and $D_{C2N}$ represent corresponding discriminators, *N* represents the noise signal data distribution, *C* represents the clean signal data distribution, *n* represents the noise signal sample, and *c* represents the clean signal sample.

## 4. Arrhythmia Detection

Presently, the most widely employed and fundamental method for examining heart diseases involves the use of the 12-lead ECG signal. It encapsulates a wealth of information related to the state of cardiac activity, serving as a crucial reference for the clinical diagnosis and treatment of cardiac morphology, heartbeat rhythm, and arrhythmia. Analyzing the waveform of the 12-lead ECG allows for a more accurate judgment of cardiac activity abnormalities.

The core of the arrhythmia diagnosis algorithm lies in automatically detecting abnormalities in the ECG waveform based on the input ECG signals. Since each input ECG sample may correspond to one or more arrhythmia events, the diagnosis algorithm should be conceptualized as a supervised multi-label classification task.

**Figure 5:**
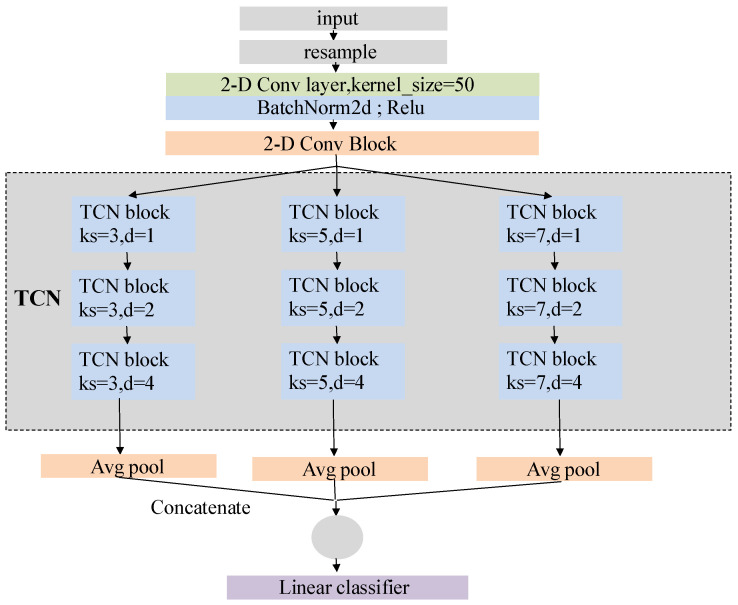
Network structure of arrhythmia detection model.

We design our model to identify various arrhythmia events, including sinus tachycardia, sinus bradycardia, sinus arrhythmia, and so forth. Figure 5 shows the comprehensive structure of the model. In particular, the network structure can be broadly categorized into four parts from top to bottom, i.e., the input layer, two-dimensional convolution and residual network layer, time convolution layer, and output layer. Referring to [37], we set the convolution kernel size to 50.

The dilated causal convolution layer in the TCN block is a crucial structure to extract temporal features. Causal convolution ensures that the operation at time *t* only uses the information before that time, as shown in Equation (5). In other words, there is no information leakage during the operation, which is consistent with the generation order of ECG sequence.

(5) $$p\left(x\right)=\prod_{t=1}^{T}p\left(X_{t}|X_{1},\ldots ,X_{t-1}\right)$$

In contrast to the regular convolution operation, dilation convolution employs a sparse sampling method that enhances a large receptive field. Equation (6) calculates the receptive field size in dilation convolution:

(6) field=(k−1)×∑d×n+1

where field is the size of receptive field, *K* is the size of convolution kernel, *D* is the expansion factor of operation, and *N* is the number of expansion causal convolution layers in the TCN block. In our model, three parallel TCN structures carry convolution kernels with lengths of 3, 5 and 7. The expansion factors of the three TCN blocks in each TCN structure are set as 1, 2 and 4, and the number of expansion causal convolution layers in the TCN blocks is two.

Thus, the receptive field size of a TCN structure with a convolutional kernel length of 3 is 29; with a kernel length of 5, the receptive field size is 57; and with a kernel length of 7, the receptive field size is 85. We conclude that in our model, the TCN structures in parallel have a maximum receptive field of 85. Furthermore, features within a distance of 57 and 29 from the current feature will be repeatedly captured, effectively increasing their weight in the final classification.

**Figure 6:**
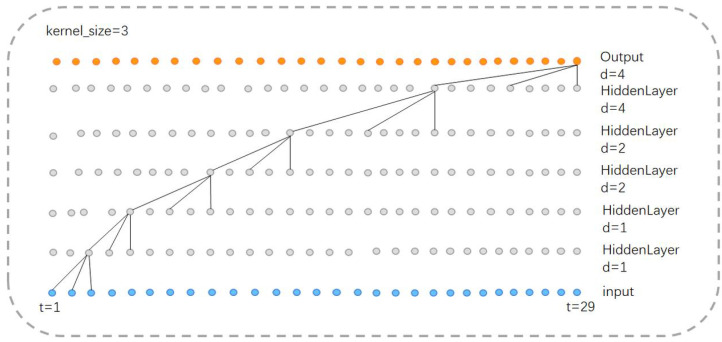
Receptive field of TCN structure (kernel_size=3).

Figure 6 illustrates the operation of the TCN structure with a convolutional kernel size of 3. The same concept applies when the kernel size is 5 or 7.

**Figure 7:**
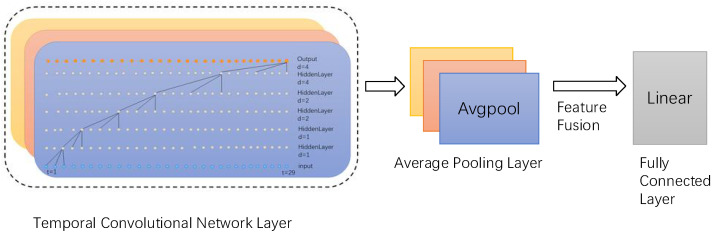
Output layer, where three colors denote three parallel layers.

In addition, the output layer of our model consists of three parallel average pooling layers and a fully connected layer (Figure 7). The high-dimensional features extracted by the TCN layer first undergo initial processing through the average pooling layer, which downsamples the high-dimensional features and reduces data dimensionality while preserving key feature information. Subsequently, such features are connected, fused, and fed into the fully connected layer. The output length of the fully connected layer is 34, equivalent to a linear classifier.

## 5. Evaluation

A. Methodology

Dataset: we use two large-scale public datasets to train and test our model, including:

- Alibaba Tianchi Dataset: This data set comes from the Engineering Research Centre of the Education Ministry of Mobile Health Management System, Hangzhou Normal University, and it contains a total of 40,000 real medical electrocardiogram samples, which are taken from patients of different age groups and genders.
- CPSC2020 Dataset [38]: This data set was collected from wearable ECG signal recording devices and contains ECG data from 10 patients with cardiovascular diseases, with each record lasting for about 24 h.

**Figure 8:**
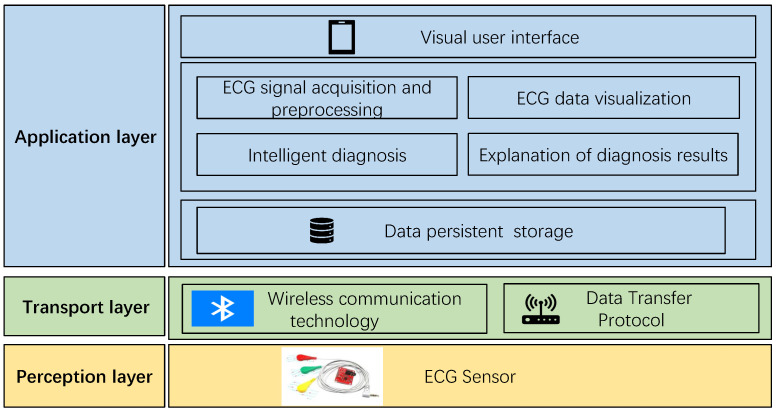
Architecture of intelligent ECG monitoring system.

From Figure 8, it is evident that, similar to the traditional architecture of IoT platforms, the overall structure of the smart home ECG monitoring system is divided into three layers: the perception layer, the transmission layer, and the application layer.

The perception layer, positioned at the bottom of the system architecture, plays a critical role in information collection. It can be likened to the “skin and senses” of the IoT, and commonly used devices in the perception layer include card readers, cameras, and sensors. In the context of this system, the perception layer refers to the ECG sensor, responsible for capturing the electrical signals generated by human heart activity. These ECG signals are then transmitted to the application layer through the transmission layer for processing and utilization.

The transmission layer functions as the channel for data transmission, employing specific data transmission protocols and wireless communication technologies. Given that the transmission layer in this system is intended for the short-range data transmission of wearable ECG sensors, low-power Bluetooth communication technology is adopted. Additionally, the data transmission protocol used is the anonymous host protocol, which will be elucidated in subsequent sections of this chapter.

**Figure 9:**
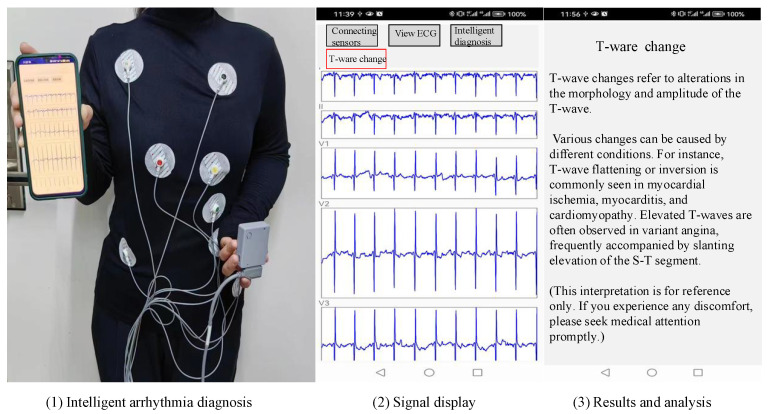
Implementation of arrhythmia diagnosis.

The application layer, also known as the processing layer, constitutes the top layer of the three-layer IoT architecture. It interfaces directly with users, providing services tailored to their needs. Serving as the bridge between the IoT system and users, the application layer closely integrates with user requirements and primarily addresses information processing, data management, and human–computer interaction. In this system, the application layer is further divided into three sub-layers: the data persistence layer, the service layer, and the visualization interface layer. The data persistence layer stores long-term data in the SQLite database provided by the Android system, supporting the service layer. The service layer implements business requirements and provides services to the interface layer. The visualization interface layer serves as an interface for direct interaction with users. The specific implementation of arrhythmia diagnosis is depicted in Figure 9.

B. Mobile Deployment

Before applying the noise reduction model, three essential steps are undertaken: model format conversion, model deployment, and model loading. Now, each step is described in detail:

- Model format conversion: The network model trained in the Python 3.8.8 environment is generally in.pth format. However, the network model supported by Android is in the .pt format. Therefore, it is necessary to use the pytorch Library in the Python environment for model format conversion. First, read the trained model into memory, and then use the method optimizer_for_mobile in the package torch.util.mobile_optimizer to converse and save the model in the .pt format.
- Mobile deployment: Mobile terminal deployment refers to the deployment of the model to the Android terminal intelligent ECG monitoring system. First, create a new assets folder in the application directory and put the format converted model into this directory. The assets directory in Android project is specially used to save various external files. The application will not process the files in this directory when compiling but will package them into. Apk files, so it is more suitable for storing model files.
- Model loading and Application: Before applying the model, the file needs to be loaded from the assets directory into memory. Then, use the load method in the Module class of the pytorch_android library to read the model and save the loaded model as a Module-type object. Finally, call the forward() method of the model object to complete inference.

C. Test of ECG Denoising Algorithm

**Figure 10:**
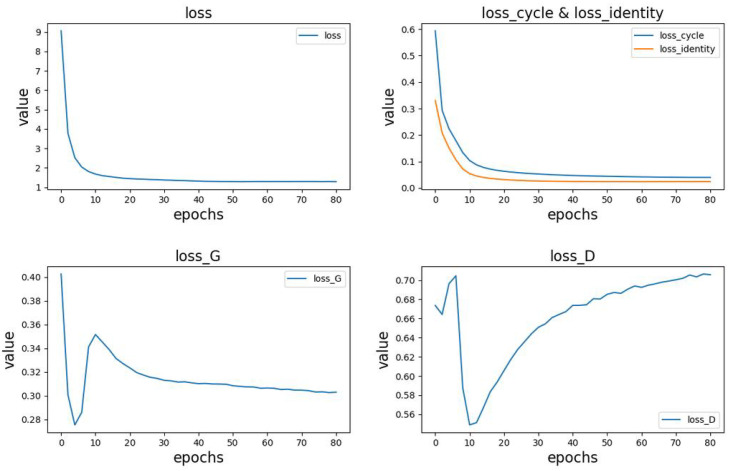
Changes in losses during training.

This article records the changes in the loss values and their components during the model training process, including the total loss (loss), generator loss (loss_G), discriminator loss (loss_D), cycle consistency loss (loss_cycle), and identity loss (loss_identity). The changes in each loss are shown in Figure 10.

From the above figure, the following observations can be made:

- The top-left position in Figure 10 represents the variation in the total loss.
- The top-right position in Figure 10 shows the trends in cycle consistency loss and identity loss. In the early stages of model training, these losses rapidly decrease and gradually converge as the training progresses. Due to pre-training of the generator, the identity loss is initially smaller than the cycle consistency loss, but their trends are similar. Additionally, since these two losses have a significant impact on the total loss, the overall trend of the total loss aligns with them.
- The bottom-left and bottom-right positions in Figure 10 represent the variations in generator and discriminator losses, respectively. Due to pre-training, the generator performs better than the discriminator in the initial stages, with lower loss values and faster reduction. During the model training process, both the generator and discriminator losses exhibit significant fluctuations, showing a fluctuating pattern. As the training progresses, the generator loss stabilizes around 0.3, while the discriminator loss stabilizes around 0.7.

**Figure 11:**
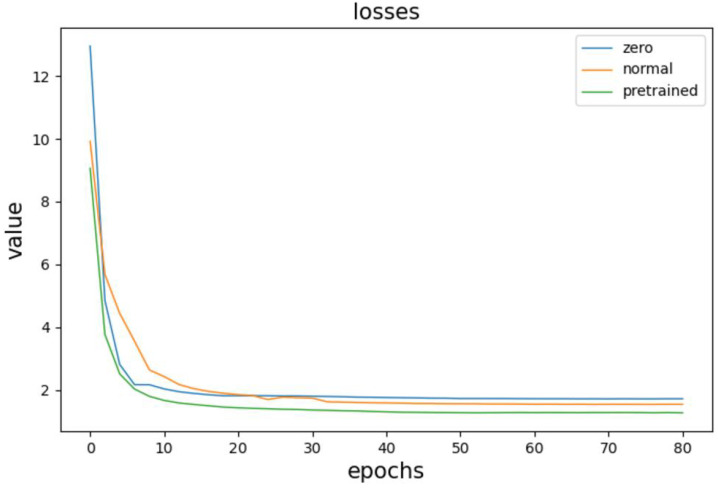
Comparative experimental results.

To verify the impact of using a pre-training strategy to initialize generator parameters in the denoising model for electrocardiogram signals, this study conducted three sets of comparative experiments. The generator parameters were initialized using random parameter initialization, normal distribution parameter initialization, and pre-training methods, respectively, and the change in total loss during model training was observed. The results of the comparisons are shown in Figure 11.

The three curves in the figure correspond to the three initialization strategies, with the bottom curve representing the loss change curve when using the pre-training method to initialize generator parameters. Comparing it with the other two curves, we can observe that when the pre-training method is used for parameter initialization, the initial value of the loss function is the smallest. The loss descends, and the model converges at the fastest rate, with the final loss value slightly smaller than the other two initialization strategies.

D. Test of System Performance

Firstly, we test the noise reduction performance of the ECG signal. To verify the denoising performance of the model, this study first added Gaussian white noise and baseline drift simulated noise signals to clean electrocardiogram signals. Such noise is consistent with the the practical noise. Although such training data is noisy, our model still works effectively with impulse noise.

**Figure 12:**
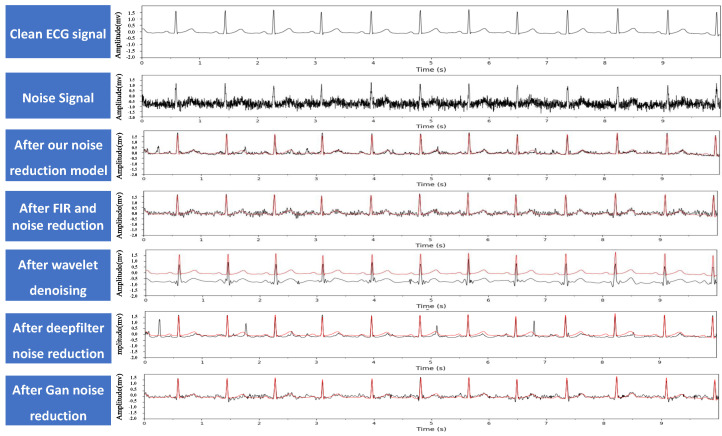
Example of ECG signal noise reduction, where the red line denotes the clean ECG signal for comparison.

Then, the proposed model was compared with commonly used traditional denoising methods, including FIR filtering [18], wavelet denoising [17], and deep learning-based denoising methods such as DeepFilter [21] and GAN [22] to denoise the noisy signals, and the denoising effects were compared. The comparison results are shown in Figure 12.

In addition, to quantitatively evaluate the denoising effects and performance of the model, this study calculated the signal-to-noise ratio (*SNR*) and mean square error (*MSE*) of the signals after denoising with different methods. *SNR* represents the ratio of the useful signal to the noise in the electrocardiogram signals. A higher *SNR* value indicates better signal quality. When the noise signal dominates, the SNR value may be negative. The specific definition of the SNR is shown in Equation (7).

(7) $$SNR=10lg\sum_{i=1}^{n}\frac{X{\left(i\right)}^{2}}{{\left[X\left(i\right)-Y\left(i\right)\right]}^{2}}$$

In the equations, *X* represents the clean electrocardiogram signal, and *Y* represents the denoised electrocardiogram signal. MSE refers to the difference between the denoised signal and the clean signal. A smaller MSE value indicates that the denoised signal is closer to the clean electrocardiogram signal, indicating a better denoising effect. The specific definition of *MSE* is shown in Equation (8).

(8) $$MSE=\frac{1}{n}\sum_{i=1}^{n}{\left[X\left(i\right)-Y\left(i\right)\right]}^{2}$$

In the equations, *X* represents the clean electrocardiogram signal, and *Y* represents the denoised electrocardiogram signal. The SNR and MSE values of different denoising models are shown in Table 1. From the data in the table, we can see that the proposed model has the highest SNR value and the lowest MSE value, indicating that its denoising performance is better than other denoising methods. Combined with the denoising effect graphs in Figure 12, it can be seen that FIR filtering and GAN [39] methods do not completely remove high-frequency noise. Wavelet denoising effectively removes high-frequency noise but cannot remove baseline drift noise, resulting in the lowest SNR value compared to the clean signal. After DeepFilter denoising, the peak position changes significantly, which may have a significant impact on the diagnostic results.

In addition, considering that the model needs to be deployed on mobile devices [7], this study compared the denoising time of different denoising methods to evaluate the real-time performance of the model. The comparison results are shown in Table 2.

From Table 2, we can see that the proposed model has the shortest denoising time. Traditional denoising methods such as FIR filtering and wavelet denoising take much longer than deep learning-based denoising methods. This is because deep learning-based methods only need to use a trained network model to complete signal denoising with one forward pass, while traditional denoising methods have a more complex computation process. In addition, the DeepFilter network has more layers and takes longer to run than the proposed model. The generator in the GAN method is an eight-layer autoencoder structure, while the proposed model is a five-layer structure. Therefore, the denoising time of the GAN method is slightly longer than that of the proposed model.

**Figure 13:**
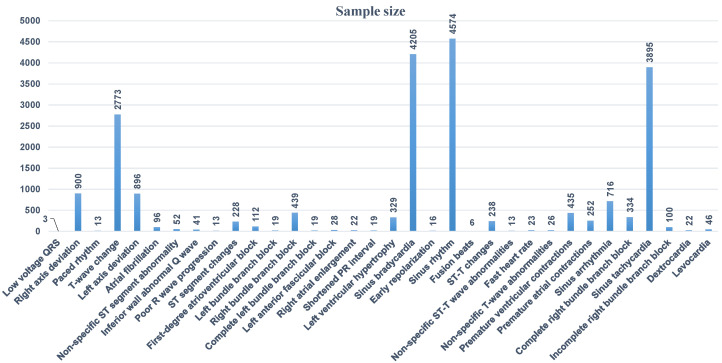
Data distribution in training data set.

Secondly, we test the arrhythmia diagnosis algorithm. As shown in Figure 13, the number of sample data of different categories in the training data set varies greatly, and the data distribution is very uneven. The samples of some common arrhythmia categories account for a large proportion in the data set, such as sinus bradycardia, sinus tachycardia, or T wave changes. The samples of some less common arrhythmia categories in clinical practice account for a small proportion in the data set, such as QRS low voltage pacing heart rate, non-specific ST segment abnormalities, etc.

To enhance the diagnostic model’s ability to identify abnormal cases comprehensively, all data were retained during the training process. However, due to significant disparities in the sample sizes across different categories, this study employed the weighted loss function BCEWithLogitsLoss as the model’s training loss function. Different weights were assigned to the loss for each category to address the issue of uneven sample distribution. The weight for each category is proportional to that category’s data in the dataset. The calculation method for the weighted loss is presented in Equation (9).

(9) $$loss_{n}=-w_{n}\left[y_{n}\cdot logx_{n}+\left(1-y_{n}\right)\cdot log\left(1-x_{n}\right)\right]$$

In the equation, *x_n_* represents the predicted value, *y_n_* represents the true value, and wn represents the class weight.

**Figure 14:**
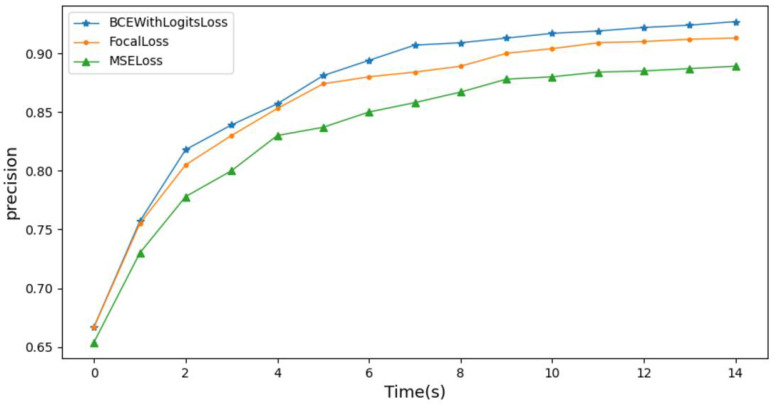
Influence of loss function on model accuracy.

To validate the impact of the loss function on the model’s accuracy, this study trained the model using three different loss functions, BCEWithLogitsLoss, FocalLoss, and MSELoss, and monitored the changes in the model’s accuracy. As shown in the comparison results in Figure 14, when training the model using the weighted loss function BCEWithLogitsLoss, the model achieved the highest diagnostic accuracy. This indicates that using BCEWithLogitsLoss can effectively address the problem of imbalanced class distribution in the training dataset and improve the accuracy of the model.

The time convolution network’s TCN layer serves as the cornerstone of the feature extraction module within the proposed arrhythmia diagnosis model. It holds the utmost significance in influencing the model’s performance. This paper explores the optimal performance of the model by adjusting the structure and parameters of the TCN layer. Refer to Table 3 for details of the model settings during the exploration process.

**Figure 15:**
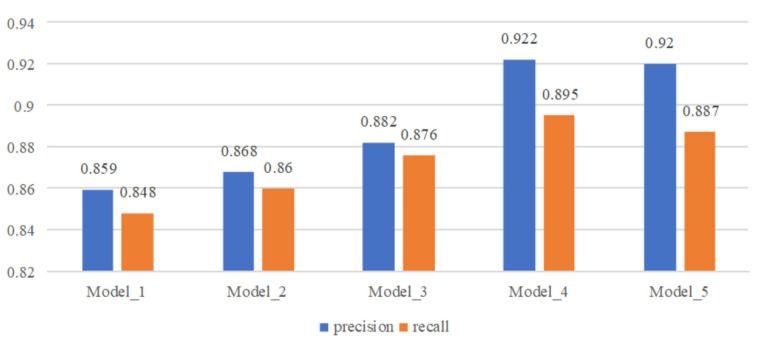
Comparison of model performance with different structures and parameters.

See Figure 15 for model performance under different network structures and parameter settings.

From the comparison results, we can see that when three parallel TCN structures are used for feature extraction, the convolution kernel size is set to 3, 5, and 7 and the expansion factor is set to 1, 2 and 4, the model performance is the best. When a single TCN structure is used for feature extraction, the performance of the model becomes better as the convolution kernel becomes larger. The parallel TCN structure can greatly improve the performance of the model. On the basis of the parallel TCN structure, when the receptive field is increased by increasing the expansion factor, the performance of the model becomes slightly worse, which may be caused by the excessive number of holes introduced in the process of feature calculation.

**Figure 16:**
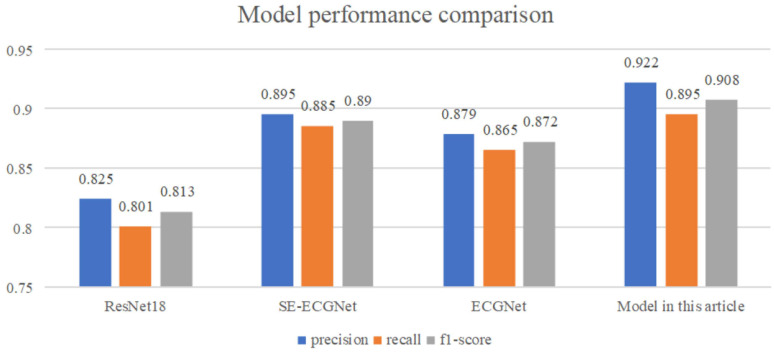
Model performance comparison.

Related works [40,41,42,43,44,45,46,47,48,49,50] and other works have shown that ResNet18 [51], SE-ECGNet [52] and ECGNet [53] are three models commonly used in the field of ECG signal detection. This paper compares the performance of the proposed model with three commonly used deep learning models for arrhythmia classification from several dimensions, including model prediction accuracy, recall rate, and F1 score. In particular, ResNet18 is based on residual connections, SE-ECGNet is based on the convolutional neural network (CNN), and ECGNet is based on the long short-term memory (LSTM) network. The mainstream solutions [37,51,54] and other works are all similarly deployed on the basis of these. The performance comparison results are shown in Figure 16.

From Figure 16, we can see that the proposed TCN-based arrhythmia diagnosis model has the best performance, outperforming the other three models in terms of model accuracy, recall rate, and F1 score. Among them, ResNet18 has the poorest performance, which may be due to the large number of sample points contained in the ECG data and the long-term sequence dependency of the data, resulting in limited ability of the network to extract temporal features. The SE-ECGNet model based on CNN extracts features using parallel two-dimensional convolution blocks and one-dimensional convolution blocks, gradually reducing the kernel size to extract features from different ranges. With a wider feature extraction range, it has better performance than ResNet18. The ECGNet model based on LSTM has slightly worse performance than SE-ECGNet, with a hidden state size of 128 and a hidden layer depth of two. The reason for the slightly poorer performance may be information loss during forward propagation, resulting in the LSTM not capturing sufficiently long ECG signal features.

**Figure 17:**
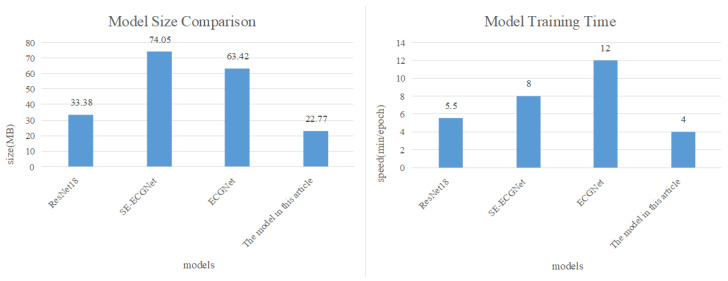
Comparison of model size and computational speed.

In addition, considering the need for mobile deployment of the model, this article compares the model from two dimensions: model size and computational speed, as shown in Figure 17.

The comparison results in Figure 17 reveal that our proposed arrhythmia diagnosis model is approximately one-third the size of SE-ECGNet and ECGNet. Additionally, in terms of computational speed, SE-ECGNet requires about 8 minutes for one round of training. Although ECGNet has a smaller scale as SE-ECGNet, its computation time is the longest due to the inability to perform parallel computations in the LSTM layer. In contrast, our proposed model demonstrates the shortest training time compared to other models. Based on these findings, we can conclude that the TCN-based arrhythmia diagnosis model is the most suitable for deployment on mobile devices, taking into account both model size and computational speed.

## 6. Conclusions

In summary, we propose a model for diagnosing arrhythmia that accurately identifies multiple common arrhythmia events and is well suited for deployment on mobile devices. Additionally, we introduce a denoising model for ECG signals to reduce the impact of noise and enhance system robustness, effectively removing mixed noise from ECG signals. Currently, our system meets the basic requirements of ECG signal acquisition and arrhythmia diagnosis in a home environment. However, our system design is not yet perfect, and there are several areas for improvement and optimization in the future:

- Conducting in-depth research on arrhythmia diagnosis algorithms: This involves incorporating information fusion methods to enhance the accuracy and reliability of the arrhythmia diagnosis.
- Performing dynamic training and optimization of the arrhythmia diagnosis model: Continuously refining and updating the model through dynamic training to adapt to evolving conditions and improve overall performance.
- Further expanding and optimizing the functionality of the system: Exploring additional features and functionalities to enhance the overall capabilities of the system, making it more comprehensive and user-friendly.

These areas of improvement and optimization will contribute to the ongoing refinement and advancement of our ECG monitoring system for home environments.

## Figures and Tables

**Table 1:** Comparison of *MSE* before and after noise reduction.

	*SNR*	*MSE*
Before noise reduction	−9.197	0.559
Model in this article	7.642	0.011
FIR filtering	6.206	0.016
wavelet denoising	−8.789	0.509
DeepFilter	3.521	0.029
GAN	4.689	0.022

**Table 2:** Noise reduction time for different models.

Model	Our Model	FIR	Wavelet	DeepFilter	GAN
Noise reduction time (ms)	12.1	292.5	53.1	21.9	13.2

**Table 3:** Model settings.

Model	TCN Structure	Convolution Kernel Size k	Expansion Factor D
Model_1	single	3	1, 2, 4
Model_2	single	5	1, 2, 4
Model_3	single	7	1, 2, 4
Model_4	paralleling	3, 5, 7	1, 2, 4
Model_5	paralleling	3, 5, 7	1, 4, 8

## Data Availability

Data are contained within the article.
